# Small Bowel Obstruction Associated With Ureterocutaneous Fistula After a Radical Cystectomy: A Case Report

**DOI:** 10.7759/cureus.52638

**Published:** 2024-01-20

**Authors:** Shintaro Arakaki, Yoji Nakamura, Hiromitsu Morioka, Hiroyuki Karimata, Mitsuhisa Takatsuki

**Affiliations:** 1 Department of Digestive and General Surgery, Graduate School of Medicine, University of the Ryukyus, Nishihara, JPN

**Keywords:** retroperitonealization, ureteral cutaneous fistula, beak sign, small-bowel obstruction, internal hernia

## Abstract

This is a case report of a man in his 60s who was diagnosed with a small bowel obstruction due to an internal hernia caused by a ureterocutaneous fistula.

Internal hernia caused by the ureter following urinary diversion is rare, posing challenges in preoperative diagnosis and carrying the risk of intraoperative injury due to the resemblance of a ureterocutaneous fistula to an adhesive band. The presentation and surgical management are discussed in this case report.

## Introduction

Radical cystectomy is a major surgical procedure primarily used to treat bladder cancer that has invaded the bladder muscle. The bladder is completely removed, urinary diversion surgery becomes necessary. This can be done in several ways, such as creating a new bladder from a segment of the intestine (neobladder) or redirecting urine through an opening in the abdomen into a urostomy bag.

It is known to be associated with some postoperative complications. While most postoperative complications are minor, up to 20% of patients experience a major complication. Gastrointestinal complications are common after radical cystectomy and urinary diversion, seen in up to 30% of patients [1]. The occurrence of a small bowel obstruction is an uncommon complication, seen in approximately 7% of patients with radical cystectomy and usually occurs secondary to internal adhesions or bowel edema [2]. Therefore, it is considered that small bowel obstruction caused by an internal hernia due to the ureter is extremely rare and difficult to diagnose.

This case report discusses a patient who developed small bowel obstruction because of a ureterocutaneous fistula after radical cystectomy.

## Case presentation

A man in his 60s presented at our hospital with chief complaints of pain in the left upper abdomen, nausea, and vomiting that had developed that afternoon. One month earlier, he had undergone right nephroureterectomy for right renal pelvis cancer, and resection of left distal ureter along with radical cystectomy for invasive urinary bladder cancer. Following that a left ureterocutaneous fistula was formed. His past medical history included hypertension and dyslipidemia.

**Figure 1:**
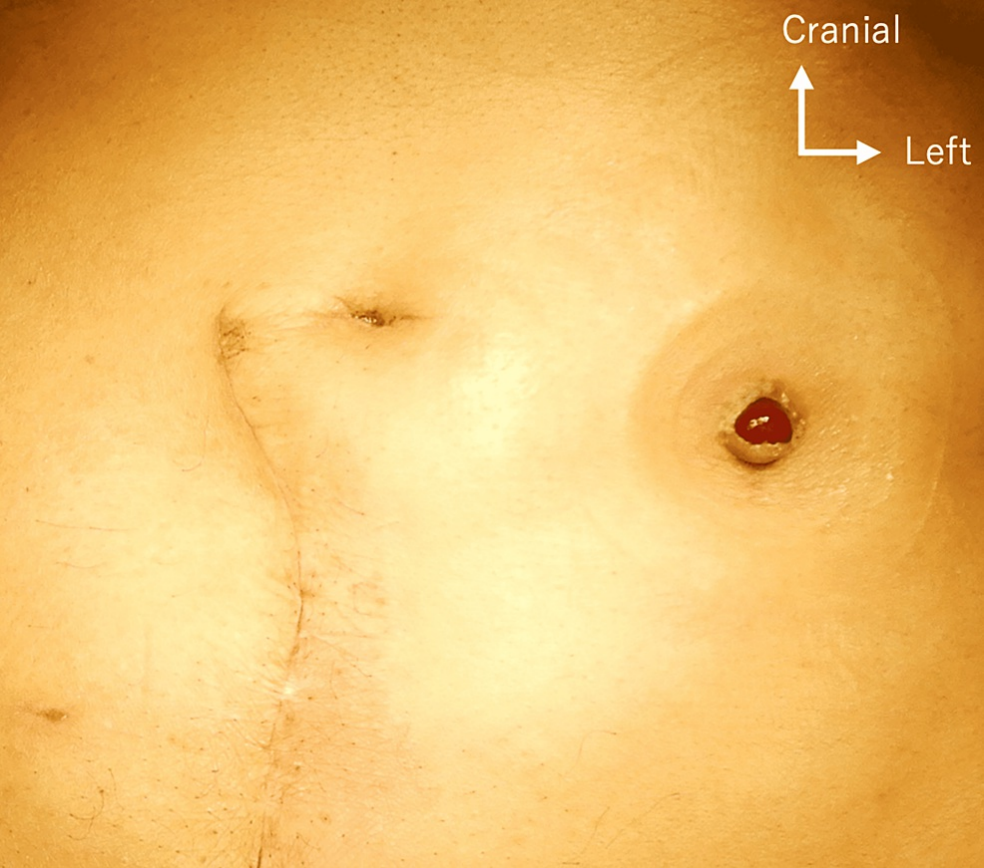
Physical findings of ureterocutaneous fistula. The patient’s ureterocutaneous fistula, which had turned dark red.

On presentation, the vital signs were within normal limits. The physical examination revealed left flank tenderness but no rebound or guarding. The ureterocutaneous fistula had turned dark red (Figure 1).

Laboratory tests showed increased creatinine (1.91 mg/dL), C-reactive protein (4.48 mg/dL), and lactate dehydrogenase (264 U/L). A blood gas analysis revealed slightly decreased pH (7.3), bicarbonate (HCO3^-^) (20.9 mmol/L), base excess (-4.8 mmol/L), and increased lactic acid (3.2 mmol/L) (Table 1).

**Table 1:** Blood test HCO3: bicarbonate

Test	Value	Reference range
Creatinine	1.91 mg/dl	0.65-1.07 mg/dl
C-reactive protein	4.48 mg/dl	0.00-0.14 mg/dl
Lactate dehydrogenase	264 U/L	124-222 U/L
pH	7.3	7.34-7.44
HCO3-	20.9 mmol/L	22-26 mmol/L
Base excess	-4.8 mmol/L	-2 - +2 mmol/L
Lactic acid	3.2 mmol/L	0.5-1.6 mmol/L

**Figure 2:**
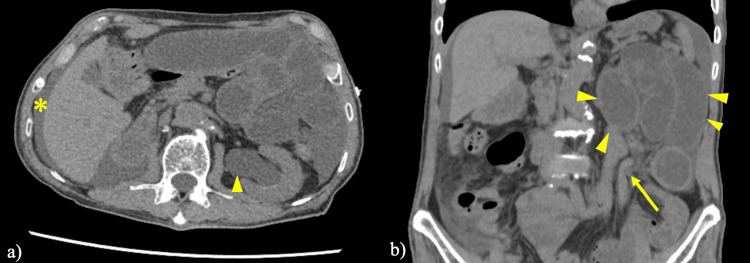
a: Abdominal axial plain CT. b: Abdominal coronal plain CT. a: Axial plain CT revealed ascites in the front of the liver (asterisk), and the hydronephrosis (arrowhead). b: Coronal plain CT showed dilated small bowel loops with the beak sign (arrowhead) and the left ureter (arrow).

The abdominal computed tomography (CT) scan showed a cluster of strangulated small bowel loops in the left flank area. Adjacent to the left ureter, there were two beak signs, which corresponded to the dual transition points of the closed-loop obstruction. The imaging also showed hydronephrosis (Figure 2A, 2B). These clinical findings were suggestive of a small bowel obstruction due to an internal hernia associated with the patient’s ureteral cutaneous fistula. We thus conducted emergency surgery.

**Figure 3:**
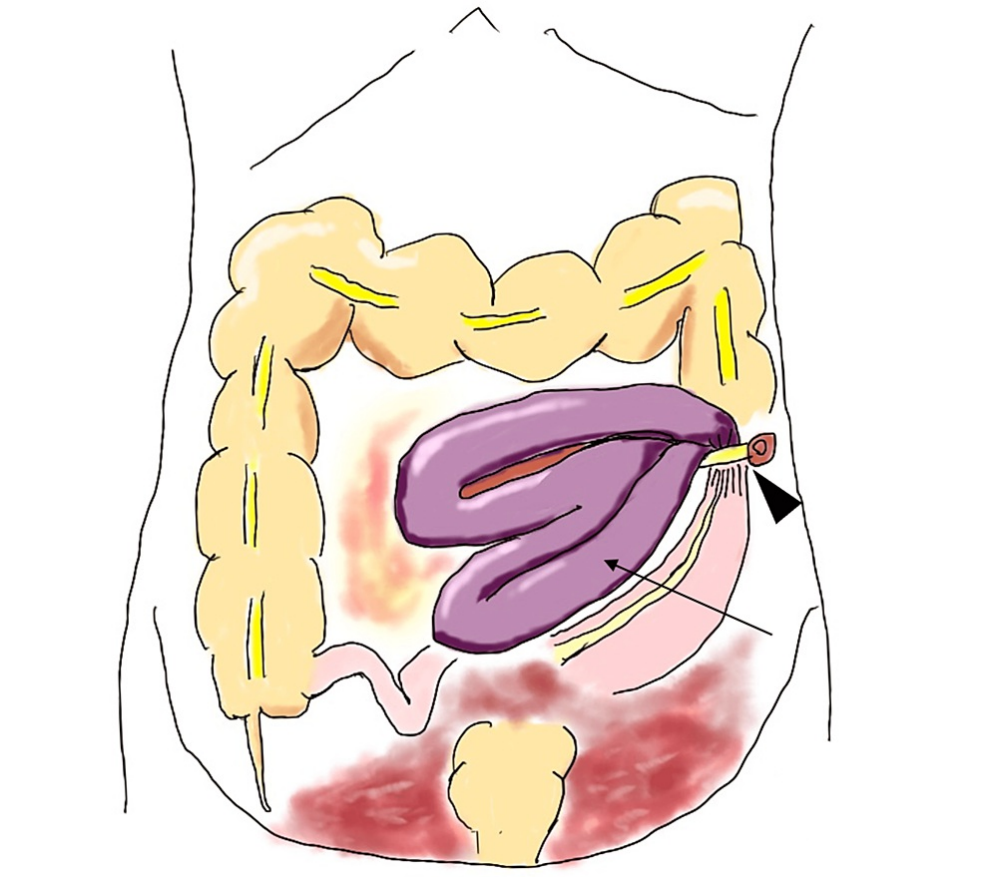
Surgical Illustration Illustration shows the relative position of the hernia loop (arrow) and ureteral cutaneous fistula (arrowhead). This illustration was drawn by Shintaro Arakaki and permission has been granted for its use in the article.

**Figure 4:**
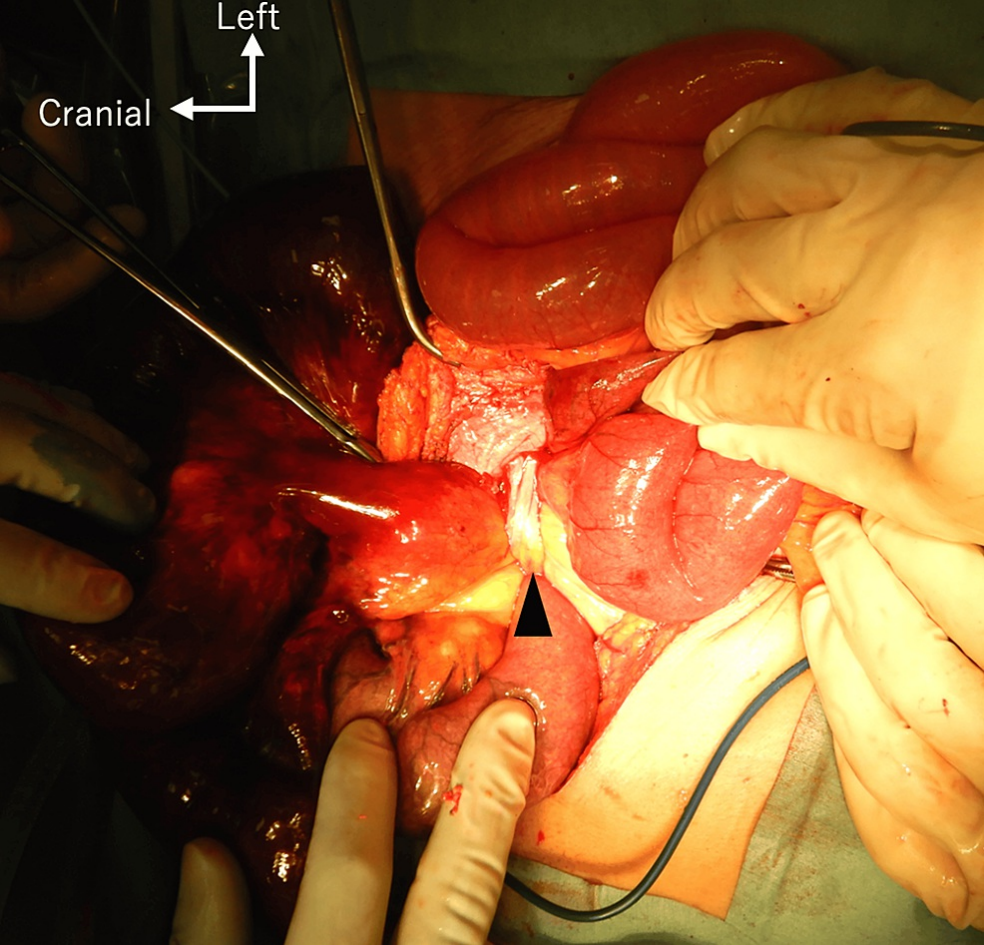
Intraoperative photograph of ureter. Figure shows that the ureter mimicked an adhesive band (arrowhead) and strangulated the small bowel.

**Figure 5:**
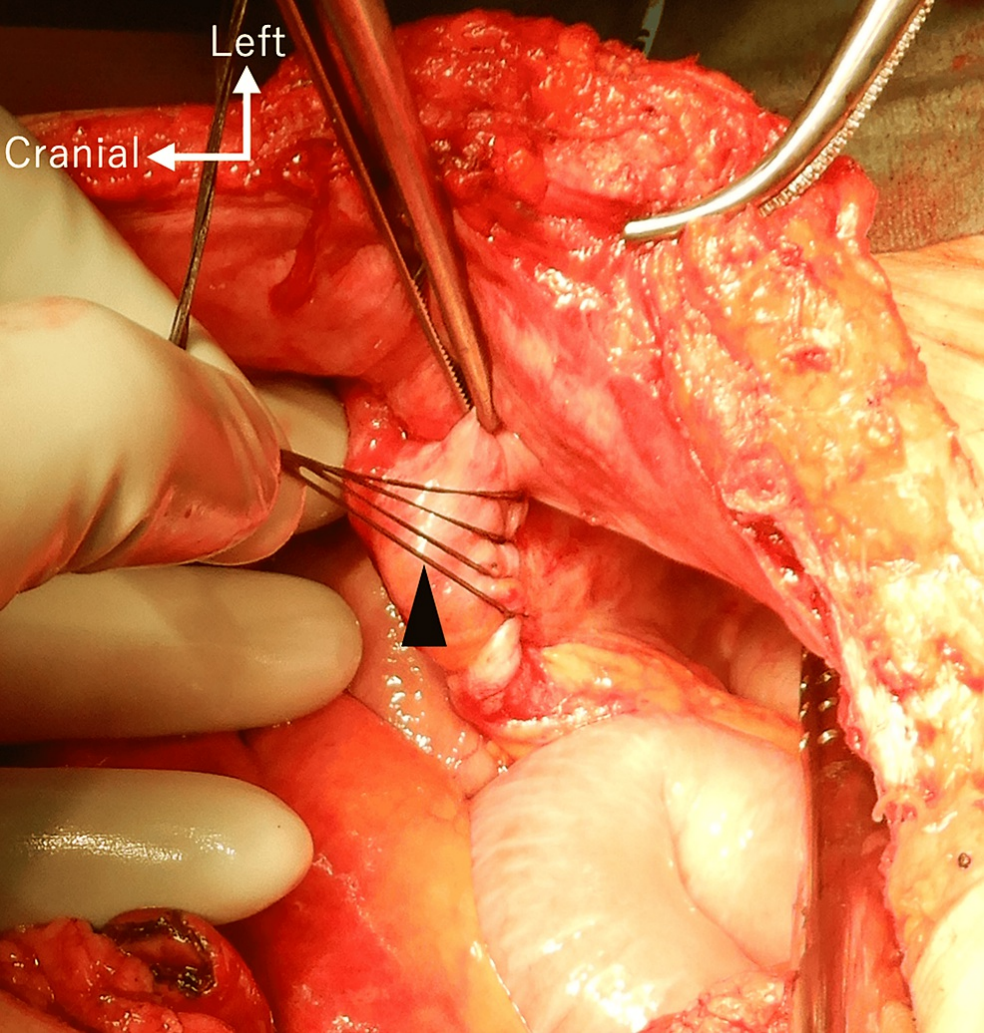
Intraoperative photograph of retroperitonealization. Figure shows retroperitonealization, which repaired of the orifice between the ureter (arrowhead) and the abdominal wall.

During the surgical procedure, it was observed that several enlarged small bowel loops were trapped in the ureterocutaneous fistula. These bowel loops were gangrenous (Figures 3, 4). There was no evidence of gastrointestinal perforation or contaminated ascites. Due to the lack of viability, the necrotic small bowel was surgically resected. We performed a functional end-to-end small bowel anastomosis. The ureter was originally retroperitonealized, but there was a partial gap, so we sutured the retroperitoneum with the abdominal wall using 3-0 V-Loc^TM^ (Covidien, Minneapolis, MI, USA) (Figure 5).

Immediate postoperative course was uneventful until a surgical-site infection developed on the ninth day after surgery. However, the patient gradually improved, and he was discharged on the 31st day after the surgery.

## Discussion

The most frequent gastrointestinal complications after urinary diversion are paralytic ileus and adhesive intestinal obstruction [1]. Small bowel obstruction due to internal herniation between ureters and the abdominal wall is extremely rare after radical cystectomy [2]. Four similar cases have been reported [3-6].To the best of our knowledge, the present case provides the first report of small bowel obstruction due to internal hernia caused by a ureteral cutaneous fistula.

The present case highlights three points that should be considered in order to prevent a ureteral injury and recurrence of an internal hernia. First, it is important to make a diagnosis preoperatively. Of course, the clinical diagnosis of an internal hernia remains extremely difficult because of its nonspecific clinical presentation and rare occurrence. In our patient’s case, we preoperatively suspected an internal hernia associated with the ureterocutaneous based on the combination of physical and imaging findings. The examination of the mucosal surface of the ureteral cutaneous fistula revealed that the fistula had turned dark red coloration, suggesting that the blood flow in the ureter was impaired. Some key image findings were also observed on CT: a cluster of strangulated small bowel loops located within the left flank, two adjacent bowel beak signs corresponding to the two transition zones of the closed-loop obstruction, the presence of ascites, and the hydronephrosis. Second, the ureter might mimic an adhesive band (Figure 4). It is important to be careful to avoid accidentally resecting the ureter. The placement of a percutaneous ureteral catheter was useful to identify the ureter in the operative field. Third, secure retroperitonealization plays an important role in the prevention of an internal hernia [3]. In our patient’s case, retroperitonealization was insufficient at the time of his cystectomy, and we thus closed the orifice between the ureter and abdominal wall during the second surgery. Not only urologists but also gastrointestinal surgeons need to become familiar with retroperitonealization.

## Conclusions

We experienced a patient with obstruction of the small intestine caused by a ureterocutaneous fistula. Although this condition is quite rare, preoperative diagnosis is important to avoid additional complications such as intraoperative ureteral injury. 
